# Severe Adverse Effects of Tirzepatide Overdose Requiring Intensive Care Unit Admission and Complex Rehabilitation

**DOI:** 10.7759/cureus.98681

**Published:** 2025-12-08

**Authors:** Mahmoud Sharafeldin, Noor Alhamdan, Ayesha Khaliq

**Affiliations:** 1 Department of Acute Medicine, St. James’s University Hospital, Leeds Teaching Hospitals NHS Trust, Leeds, GBR

**Keywords:** glp1 receptor agonist, mounjaro, overdose, tirzepatide, weight loss

## Abstract

Tirzepatide, a dual glucagon-like peptide-1 (GLP-1)/glucose-dependent insulinotropic polypeptide receptor agonist, is increasingly prescribed privately in the United Kingdom for weight loss. While gastrointestinal side effects are well recognized, severe metabolic derangements and hematological complications remain rarely documented. We report the case of a 39-year-old man with no significant comorbidities who developed life-threatening hypoglycemia, profound electrolyte abnormalities, pancytopenia, and aspiration pneumonia following unsupervised rapid dose escalation of tirzepatide used for weight loss. His clinical course was complicated by respiratory failure, septic shock, multiorgan dysfunction, and prolonged intensive care unit stay requiring mechanical ventilation, renal replacement therapy, tracheostomy, and complex neurorehabilitation. This case highlights the critical complications associated with inappropriate tirzepatide dosing and underscores the need for clinician vigilance and patient education as GLP-1 receptor agonist use continues to rise in the community.

## Introduction

The use of glucagon-like peptide-1 receptor agonists (GLP-1 RAs), particularly tirzepatide, for weight management is rapidly increasing in the United Kingdom. According to estimates from online pharmacies in the United Kingdom, as many as 500,000 people may be using Mounjaro® or Wegovy® via private online prescriptions in the United Kingdom, and, in selected pharmacies, Mounjaro accounts for approximately 70% of their GLP-1 analog sales [1]. While gastrointestinal adverse effects of GLP-1 RAs are well recognized, severe metabolic disturbances, hematological complications, and critical illness remain rare and underreported. Unsupervised use and inappropriate dose escalation pose particular risks. We present a case of tirzepatide overdose leading to profound metabolic derangement, bone marrow suppression, multiorgan failure requiring prolonged intensive care unit (ICU) admission, and complex rehabilitation.

This article was presented as a poster at the Society of Acute Medicine conference in Manchester, UK (SAMManchester) on September 22-23, 2025.

## Case presentation

A 39-year-old man with a body mass index of 34.8 kg/m² and no other significant comorbidities had been self-administering tirzepatide for weight loss, escalating doses without clinical supervision. His regimen was initiated at 2.5 mg weekly with 2.5 mg increments every four weeks. After missing the 10 mg dose for a month due to delivery issues, he resumed at 12.5 mg. Four days later, he was found by his mother on the floor in a confused state, drifting in and out of consciousness. Emergency services were contacted, and he was transferred to the hospital.

Upon arrival at the emergency department, his vital signs were a respiratory rate of 24 breaths per minute, oxygen saturation of 96% on room air, body temperature of 35.2°C, blood pressure of 108/66 mmHg, and a heart rate of 59 beats per minute. His Glasgow Coma Scale (GCS) score was 9 out of 15.

Laboratory investigations, as shown in Table 1, revealed pancytopenia (hemoglobin: 82 g/L; white cell count: 1.76 × 10⁹/L; platelets: 30 × 10⁹/L), macrocytosis (mean corpuscular volume: 123 fL), undetectable serum folate (<1 µg/L), coagulopathy (international normalized ratio: 3), and elevated lactate dehydrogenase (512 IU/L). Electrolytes were significantly deranged (potassium: 2.6 mmol/L; magnesium: 0.56 mmol/L; phosphate: 0.39 mmol/L). Liver function tests were impaired (bilirubin: 74 µmol/L; alanine aminotransferase: 413 U/L). Venous blood gas analysis revealed a high anion gap metabolic acidosis (pH: 7.181; bicarbonate: 11.5 mmol/L; ketones: 3.9 mmol/L; anion gap: 21.2). Capillary glucose was 1.5 mmol/L.

**Table 1:** Laboratory findings. MCV = mean corpuscular volume; BBV = blood-borne virus; HIV = human immunodeficiency virus; HBV = hepatitis B virus; HCV = hepatitis B virus; INR = international normalized ratio; LDH = lactate dehydrogenase; ALT = alanine aminotransferase; ALP = alkaline phosphatase; VBG = venous blood gas

Test	Result	Reference range	Units
Hemoglobin	82	135–180	g/L
White cell count	1.76	4.0–11.0	×10⁹/L
Platelets	30	150–400	×10⁹/L
MCV	123	78–100	fL
Serum folate	<1	5.4–24.0	µg/L
Vitamin B12	249	211–911	ng/L
Iron	20	11.6–31.3	µmol/L
Vitamin D (25-OH)	89.4	50–125	nmol/L
Copper	8.8	11–22	µmol/L
Selenium	0.33	0.80–2.00	µmol/L
Zinc	4.7	9.8–17.9	µmol/L
BBV screen (HIV, HBV, HCV)	Negative	–	–
INR	3.0	0.8–1.3	–
LDH	512	120–246	IU/L
Potassium	2.6	3.5–5.3	mmol/L
Magnesium	0.56	0.70–1.00	mmol/L
Bilirubin (total)	74	2–21	µmol/L
ALT	413	<40–50	U/L
ALP	46	30–130	U/L
Procalcitonin	1.7 → 136	<0.05–0.1	ng/mL
Venous pH	7.181	7.35–7.45	–
Bicarbonate (VBG)	11.5	22–29	mmol/L
Ketones (blood)	3.9	<0.6	mmol/L
Anion gap	21.2	8–16	mmol/L
Capillary glucose	1.5	3.5–6.0	mmol/L

His GCS score improved to 14/15 following correction of hypoglycemia, and more history was obtained. His oral intake had been markedly reduced; he was “barely eating” for several days with profound loss of appetite. He denied any alcohol or substance use and was not following a specific diet. He rapidly deteriorated, and on his second day of admission, he developed increasing oxygen requirements and cardiovascular collapse, requiring ICU admission later on. Blood cultures grew *Staphylococcus aureus* on day four of admission, sputum cultures grew *Escherichia coli*, and viral screening in the ICU was positive for metapneumovirus, as shown in Table 2. His procalcitonin was 1.7 ng/mL on day four of admission, and two days later, it increased to 136 ng/mL in the ICU following the development of severe sepsis.

**Table 2:** Microbiology results. PCR = polymerase chain reaction; MRSA = methicillin-resistant *Staphylococcus aureus*

Test	Result	Interpretation
Blood cultures	Staphylococcus aureus	Pathogenic; consistent with bacteraemia/sepsis
Sputum culture	Escherichia coli	Pathogen isolated; supports aspiration pneumonia
Viral PCR screen	Metapneumovirus detected	Respiratory viral infection present
MRSA screen	Negative	No colonization
COVID-19 PCR	Negative	No SARS-CoV-2 infection
Urine culture	No growth	No urinary infection

**Figure 1:**
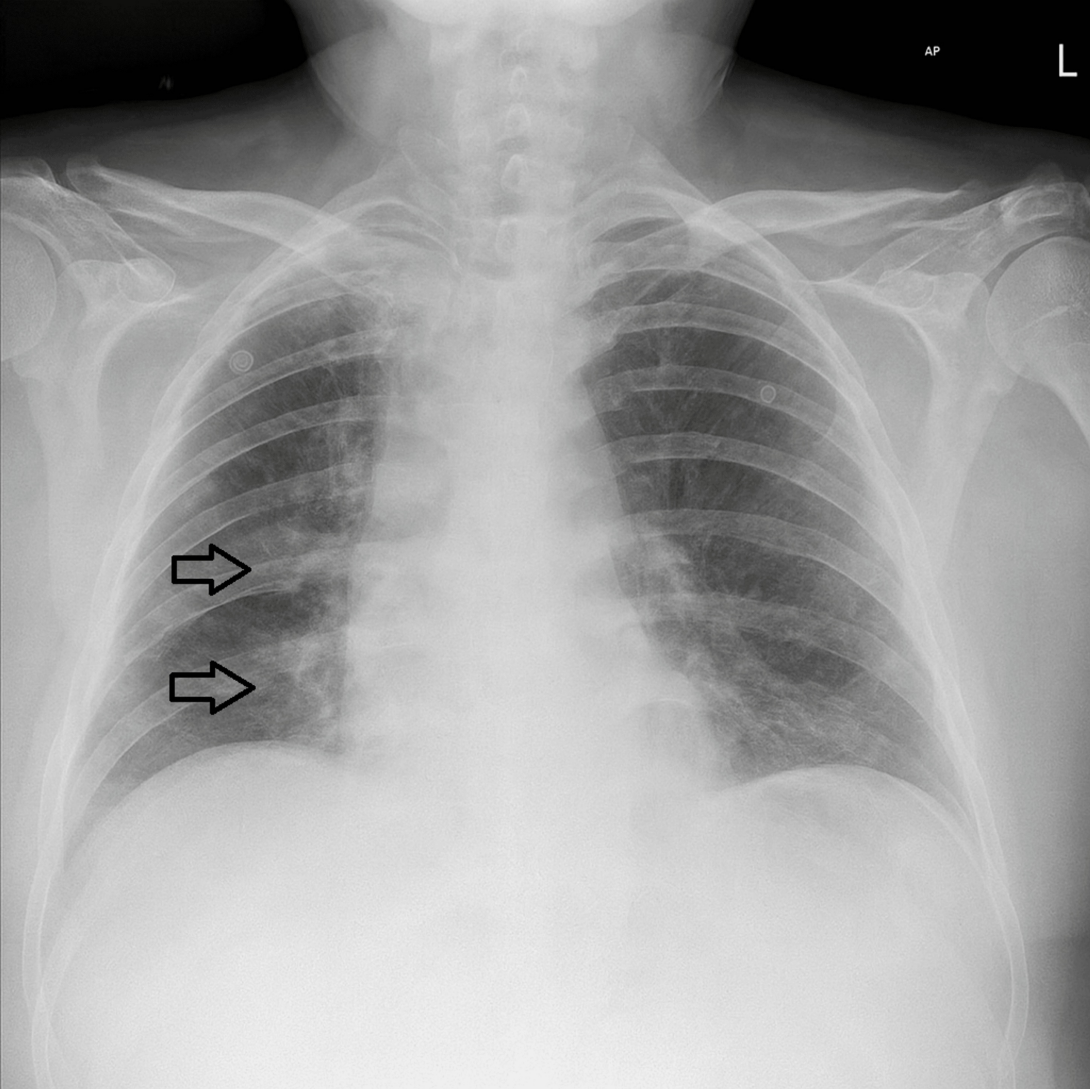
Chest X-ray showing right-sided mid-to-lower zone air-space opacification, consistent with aspiration pneumonia.

**Figure 2:**
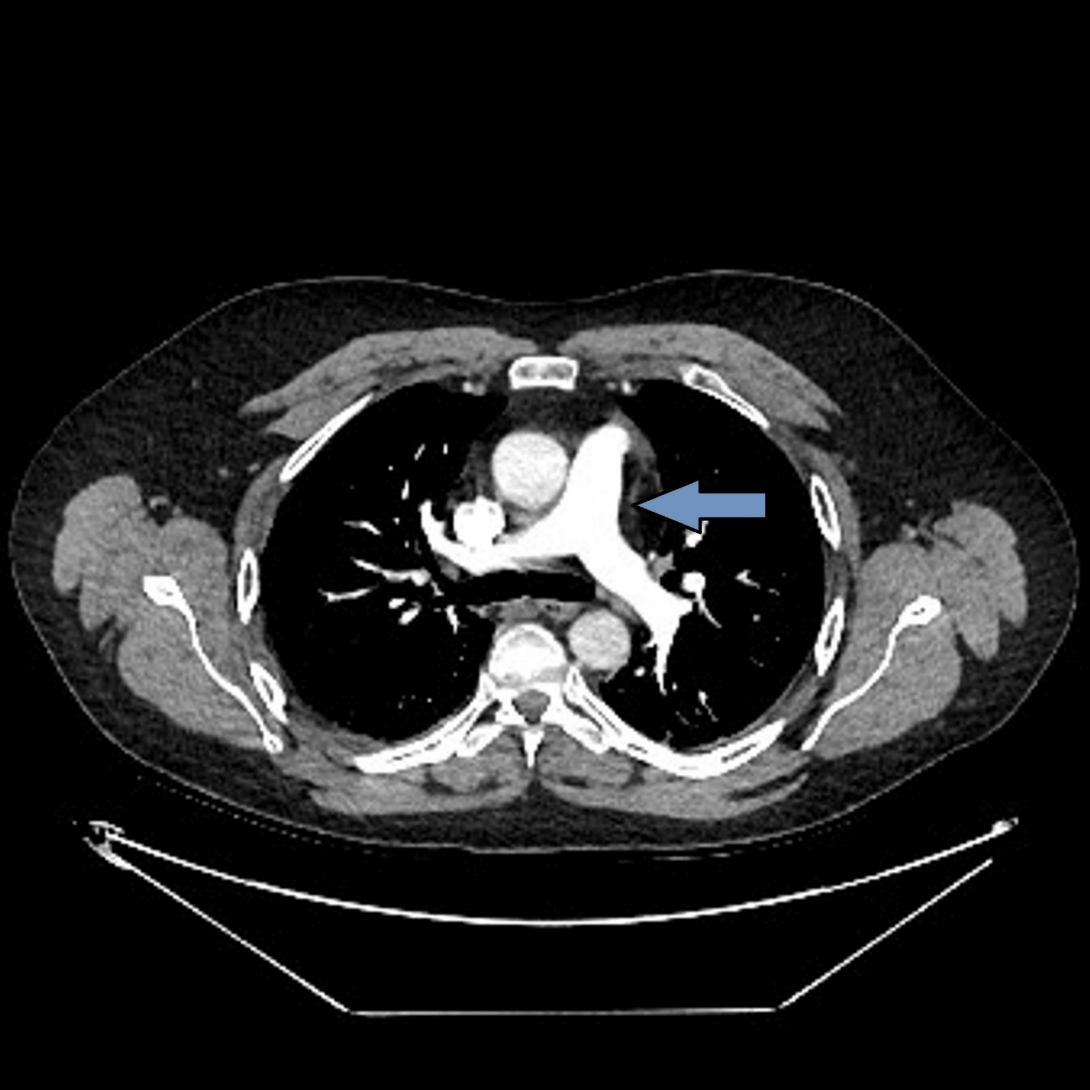
CT pulmonary angiogram axial image demonstrating well-opacified main and proximal pulmonary arteries with no intraluminal filling defects, ruling out central pulmonary embolism.

Admission chest radiography showed right-sided mid-to-lower zone air-space opacification, consistent with aspiration pneumonia, as shown in Figure 1. CT pulmonary angiography excluded pulmonary embolism, as shown in Figure 2. Bedside transthoracic echocardiography in the ICU demonstrated severe right ventricular dilatation with tricuspid annular plane systolic excursion of 1.7 cm and severely impaired radial functions with a hyperdynamic left ventricle related to sepsis and critical illness. During rehabilitation, electromyography showed severe sensory-motor neuropathy consistent with critical illness neuropathy, as shown in Table 3.

**Table 3:** Electromyography findings suggesting a severe degree of generalized sensory motor neuropathy of axonal loss pattern which is length dependent in nature, possibly secondary to critical illness neuropathy. Fib = fibrillation potentials; PSW = positive sharp waves; Amp = amplitude; Dur = duration; PPP = polyphasic potentials; IP = interference pattern

Muscle	Nerve	Roots	Fib	PSW	Other	Amp	Dur	PPP	IP	Firing
Right tibialis anterior	Deep peroneal (fibular)	L4–L5	2+	2+	None	1+	2+	2+	Reduced	Fast
Right vastus medialis	Femoral	L2–L4	2+	2+	None	1+	2+	2+	Reduced	Fast
Left vastus medialis	Femoral	L2–L4	1+	1+	None	1+	2+	2+	Reduced	Fast
Right extensor digitorum communis	Radial	C7–C8	1+	1+	None	1+	1+	1+	Reduced	Fast
Right deltoid	Axillary	C5–C6	2+	2+	None	1+	1+	1+	Reduced	Fast
Right trapezius (upper)	Accessory (spinal)	C3–C4	None	None	None	N	N	N	N	N

Management included immediate cessation of tirzepatide, 10% dextrose infusion, intravenous Pabrinex to cover for Wernicke’s encephalopathy, broad-spectrum antibiotics, nasogastric enteral feeding, electrolyte replacement guided by National Institute for Health and Care Excellence refeeding recommendations [2], intravenous folinic acid, vitamin K replacement, and blood product transfusions, including platelet transfusion to facilitate therapeutic anticoagulation pending pulmonary angiography, and red cell transfusions for severe sepsis-related anaemia, guided by the local hematology team’s advice.

The patient required a prolonged ICU admission for respiratory failure, aspiration pneumonia, septic shock, and multiorgan dysfunction. Over his 25-day ICU stay, he required intubation, mechanical ventilation, vasopressors, renal replacement therapy, and tracheostomy before stepping down to a medical ward. He subsequently required complex multidisciplinary rehabilitation, including speech and language therapy for dysarthria, neurorehabilitation for critical illness polyneuropathy, and dietetic support.

## Discussion

Tirzepatide is a dual glucagon-like peptide-1 (GLP-1) and glucose-dependent insulinotropic polypeptide (GIP) receptor agonist that enhances glucose-dependent insulin secretion, suppresses glucagon, and slows gastric emptying, making it highly effective for weight loss and type 2 diabetes management [3-5]. Although the safety profile in clinical trials has been dominated by gastrointestinal symptoms such as nausea, vomiting, and diarrhea, post-marketing data increasingly describe infrequent but serious metabolic complications, particularly when these drugs are used off-label or without medical supervision [6-8]. In our patient, rapid unsupervised dose escalation, treatment interruption, and re-initiation at a higher dose occurred in the context of obesity without diabetes, creating a high-risk scenario for severe adverse effects. While hypoglycemia risk is highest in diabetic patients on insulin or sulfonylureas, profound caloric restriction, starvation ketosis, and overdose can precipitate hypoglycemia in non-diabetic individuals as well.

Recent post-marketing data have begun to characterize tirzepatide-related ketoacidosis and hypoglycemia in non-diabetic individuals. Iqbal et al. reported the first case of tirzepatide-induced ketoacidosis in an obese non-diabetic patient, in whom starvation ketosis and impaired gluconeogenesis were implicated as key mechanisms [9]. A subsequent case series by Bitar et al. described hypoglycemic ketoacidosis in several non-diabetic patients on tirzepatide for weight loss, again attributing the metabolic decompensation to reduced oral intake and glycogen depletion exacerbated by enhanced insulin secretion [10]. More recently, Huang et al. conducted a retrospective pharmacovigilance analysis of tirzepatide adverse drug reactions and highlighted that serious endocrine and metabolic events, including ketoacidosis, tend to occur within the first six months of therapy, underlining the importance of early monitoring and dose titration [11]. Compared with these reports, our case is distinguished by the combination of profound hypoglycemia, starvation ketoacidosis, severe electrolyte derangement, and multiorgan failure requiring prolonged ICU admission.

Serious metabolic decompensation has also been reported with other GLP-1 receptor agonists. Case reports describe diabetic or euglycemic ketoacidosis following dulaglutide and semaglutide, often after insulin reduction or discontinuation or in the context of prolonged vomiting and reduced caloric intake [12]. Pharmacovigilance analyses and regulatory safety communications, including the UK Medicines and Healthcare products Regulatory Agency Drug Safety Update, have similarly drawn attention to diabetic ketoacidosis events when GLP-1 RAs are used with rapid changes in concomitant insulin therapy [13]. Collectively, these data support the concept that GLP-1 RA-associated ketoacidosis is typically precipitated by a combination of appetite suppression, reduced carbohydrate intake, dehydration, and inappropriate adjustment of other glucose-lowering agents rather than a direct toxic effect. Our case extends this literature by demonstrating that in a non-diabetic patient using tirzepatide solely for weight loss, extreme anorexia and caloric restriction can lead not only to ketoacidosis and hypoglycemia but also to severe micronutrient deficiency and bone-marrow suppression.

The hematological abnormalities in this patient, pancytopenia with macrocytosis and undetectable folate, are consistent with megaloblastic marrow failure secondary to severe folate deficiency. Several recent case reports have described profound pancytopenia due to isolated folate deficiency or combined folate and vitamin B12 deficiency, with rapid hematological recovery following replacement therapy [14]. Although these reports involve diverse etiologies (alcohol misuse, malabsorption, medication-induced deficiency), the final common pathway of ineffective erythropoiesis and cytopenia mirrors the pattern observed in our patient. In our case, there was no evidence of marrow infiltration or primary hematological malignancy, and cytopenias improved with folinic acid, nutritional rehabilitation, and resolution of sepsis, supporting the interpretation that severe nutritional deficiency from tirzepatide-induced anorexia was the primary driver. Upon hospital admission, there were no initial clinical or biochemical features of sepsis. He subsequently developed sepsis during his hospital course, likely facilitated by severe pancytopenia and bone marrow suppression.

Emerging observational data suggest that nutritional deficiencies may be more common than initially appreciated in patients treated with GLP-1 RAs. Butsch et al. reported that in a large cohort of adults with type 2 diabetes receiving GLP-1 RAs, clinically diagnosed nutritional deficiencies occurred in approximately 12.7% of patients within six months and over 22% within a year, with folate and vitamin D among the most frequently affected micronutrients [7]. While this study did not specifically evaluate tirzepatide overdose or unsupervised use, it reinforces the concept that appetite suppression and altered intake associated with GLP-1 RA therapy can have clinically relevant nutritional consequences. Our case illustrates an extreme manifestation of this risk, in which prolonged poor intake led not only to micronutrient deficiency but also to profound functional decline, critical illness neuropathy, and complex rehabilitation needs.

Finally, the neurological complications observed in our patient, severe critical illness polyneuropathy and prolonged weakness, are more plausibly linked to ICU-acquired weakness, sepsis, multiorgan failure, and immobility than to a direct neurotoxic effect of tirzepatide. Systematic reviews suggest that GLP-1 RAs may, in fact, exert neuroprotective effects in diabetes-related neurological complications, although recent pharmacovigilance analyses have identified various neurological adverse events temporally associated with GLP-1 RAs, emphasizing the need for continued surveillance [11]. Our case underscores that when GLP-1/GIP agonists precipitate critical illness through severe metabolic derangement, the downstream neurological sequelae can be substantial, even if indirect.

## Conclusions

This case is among the few cases in the literature describing life-threatening, ICU-level complications of tirzepatide overdose and highlights the potential for critical illness. With private prescribing on the rise and unregulated dose escalation common, acute medical teams are increasingly encountering patients with adverse effects from the unsupervised and inappropriate use of these agents. This case highlights several important learning points. First, unsupervised dose escalation and re-initiation of GLP-1 RAs at higher doses can precipitate life-threatening hypoglycemia, ketoacidosis, and electrolyte disturbances. Second, profound appetite suppression can lead to severe nutritional deficiencies, including folate-deficiency pancytopenia, with significant hematological and functional consequences. Third, given accumulating evidence of ketoacidosis, nutritional deficiency, and other serious adverse events with GLP-1 RAs, clinicians should provide clear counseling on safe use, screen for red-flag symptoms, monitor nutritional status, and maintain a low threshold for hospital assessment in patients using these agents for weight loss outside structured diabetes care pathways.
